# Family Health Conversations—A Short-Term Supportive Intervention to Improve Family Well-Being, Functioning, and Involvement in Care After Open-Heart Surgery: A Multicenter, Randomized, Parallel-Group Superiority Trial

**DOI:** 10.1177/10748407261440159

**Published:** 2026-04-22

**Authors:** Anna Drakenberg, Daniel R. Smith, Ann-Sofie Sundqvist, Christine Leo Swenne, Elisabeth Ericsson

**Affiliations:** 1 School of Health Sciences, Faculty of Medicine and Health, Örebro University, Sweden; 2 Department of Cardiothoracic and Vascular Surgery, Örebro University Hospital, Sweden; 3 School of Medical Sciences, Faculty of Medicine and Health, Örebro University, Sweden; 4 Department of Public Health and Caring Sciences, Uppsala University, Sweden

**Keywords:** cardiovascular surgical procedures, family health conversations, family nursing, health-related quality of life, randomized controlled trial, sense of coherence

## Abstract

In this study, a nurse-led supportive family health conversation intervention delivered through one to three video-conferencing sessions was evaluated for patients undergoing open-heart surgery and their self-selected family members. Based on the Family Systems Nursing framework, the intervention aimed to improve family well-being, functioning, and involvement by fostering shared understanding and challenging limiting beliefs. Patients and family members were randomized into two groups. Both received usual surgical care, while the intervention group also participated in digital family health conversations before and after surgery. Participants completed questionnaires at baseline and at 30 and 90 days after discharge. The analysis included 101 patients (control = 54, intervention = 47) and 99 family members (control = 52, intervention = 47). The intervention was not superior to usual care for the primary outcome, family well-being. Most secondary outcomes showed no effect, although some aspects of quality of life improved. Further research should examine long-term effects, feasibility, and appropriate outcome measures.

**Clinical trials register number and URL:** NCT05045196, https://clinicaltrials.gov/study/NCT05045196?cond=NCT05045196&rank=1;

## Introduction

Families play a significant role in the care of patients both before and after open-heart surgery (Bjørnnes et al., 2019). Importantly, however, the family itself has care needs that must be addressed. Elective open-heart surgery can impose significant physical and psychological strain on individuals undergoing the procedure (Karlsson et al., 2005; Kemp et al., 2020). Patients often rely on their family members to help them remember information (Drakenberg et al., 2024), and family members often assume responsibility for the practical rehabilitation process at home (Drakenberg et al., 2025). This is a role for which they may not be adequately prepared (Bjørnnes et al., 2019). Prior to surgery, the fear of death and adverse events is noticeable for patients and their family members. Seeing the sadness of one’s family members has been described as being more painful than surgery itself (Drakenberg et al., 2024).

In high-income countries, the majority of individuals who undergo open-heart surgery are men older than 65 (Lai et al., 2024). Women tend to undergo the surgery at a greater age, frequently report more severe symptoms prior to surgery (Cho et al., 2021), and experience poorer outcomes after such procedures than men do (Cho et al., 2021; Lai et al., 2024). Postoperatively, the majority of family members who assume the primary role in supporting patients’ rehabilitation at home are women (Bjørnnes et al., 2019).

Open-heart surgery is indicated for numerous conditions, including congenital heart disease and acute myocardial infarction (Neumann et al., 2019; Vahanian et al., 2022). Consequently, patients’ symptom profiles prior to surgery may differ. For example, some patients who undergo valve replacement are asymptomatic, whereas others suffer from heart failure following myocardial infarction (Neumann et al., 2019; Vahanian et al., 2022). In addition to receiving individualized care, these patients receive standardized open-heart surgical care, including similar preoperative information and high-tech postoperative care in a cardiothoracic intensive or intermediate postoperative care unit (Grant et al., 2024). The recovery period at home typically lasts 6 to 8 weeks. During this time, patients are on sick leave and are advised to avoid heavy lifting (Mortensen et al., 2022).

In addition to encouraging and preserving social support for patients undergoing open-heart surgery (Drakenberg et al., 2024) and their family members (Drakenberg et al., 2025), a need for professional support has been identified (Bjørnnes et al., 2019; Drakenberg et al., 2025). A variety of social support interventions are currently in use within the context of cardiovascular care. However, evidence of the relative efficacy of these interventions is inconclusive (Clayton et al., 2019; Purcell et al., 2023). It has been proposed that family-inclusive social support interventions may be the optimal approach in this context (Clayton et al., 2019). The family health conversation intervention model (Benzein et al., 2008) is grounded in the Family Systems Nursing framework, which views health and illness as affecting the entire family (Shajani & Snell, 2023). The intervention originates from the so-called Calgary models (Shajani & Snell, 2023), which were adapted and further developed for Swedish patients and their families. The family health conversation model was designed to promote family health and alleviate illness and suffering for the entire family and has 12 core components (Östlund et al., 2015; Östlund & Persson, 2014) as presented along with the proposed mechanisms of change and expected outcomes, as shown in Table 1. By engaging in family storytelling, verbalizing illness beliefs, and reflecting on the need for altered health behaviors, families can identify strategies to improve their health, reach a communal understanding, and change constraining illness beliefs (Benzein et al., 2008; Persson & Benzein, 2014). Nurses are historically well-positioned to participate in family health conversations, combining clinical expertise with a systemic perspective and substantial patient contact, which creates natural opportunities for therapeutic conversations (Shajani & Snell, 2023). In Sweden, university courses are critical for the development of nursing competencies in family nursing and family health conversations, and the nurse who participated in the intervention in this study possessed this advanced training. While the emphasis on the role of nursing within the theoretical framework has evolved over time, internationally, workshops in advanced family systems care are offered to practicing health care professionals by the original authors of the Calgary models and the illness belief model (Bell & Wright, 2015), expanding the reach and application of Family Systems Nursing beyond traditional nursing settings.

**Table 1.** Techniques and Theoretical Assumptions Regarding the Working Mechanisms and Expected Outcomes of the Family Health Conversations (FamHC).

Treatment techniques (Östlund et al., 2015)	Working mechanisms (Persson & Benzein, 2014)	Outcomes (Benzein et al., 2008; Persson & Benzein, 2014)
**Core components of the FamHC** 1. Reflect with the family on expectations of the conversation series. 2. Examine the family’s structure. 3. Ensure that each family member has space in the conversations and the opportunity to share their experiences. 4. Collaboratively prioritize the issues that need the most attention. 5. Explore important elements of the family’s narratives. 6. Use reflective questions 7. Pose appropriately unusual or challenging questions to gently question family beliefs. 8. Offer praise and acknowledge suffering. 9. Invite family members to consider and reflect on each other’s stories. 10. Provide the nurses’ own reflections. 11. Ask about developments since the previous conversation. 12. Conclude the conversation series during the final meeting, followed by the delivery of a closing letter from the nurse to the family.	Cocreated alternative explanations Improved understanding Visualization of various beliefs Constructing a collective narrative	Improved family health Communal understanding Changing constraining beliefs

The concept of health involves not only the absence of illness but also physical, mental, and social well-being (World Health Organization [WHO], 2020). In this study, family well-being was operationalized through the measurement of family sense of coherence (Antonovsky & Sourani, 1988; Möllerberg et al., 2020), family hardiness (Persson et al., 2016), health-related quality of life (Ohlsson-Nevo et al., 2021), and patients’ physical, mental, and social postoperative recovery profile (Allvin et al., 2011). A sense of coherence is a concept that reflects the extent to which individuals perceive their world as comprehensible, manageable, and meaningful (Antonovsky, 1987). The concept was later applied to family life, with a high family sense of coherence scale score indicating a potential buffer against life stressors (Antonovsky & Sourani, 1988). Family hardiness refers to an individual’s perception of his or her family’s ability to manage conflict (Persson et al., 2016) and, like the concept of sense of coherence, represents a measure of stress management in line with stress-buffering theory (Cohen et al., 2000). In this study, health-related quality of life is defined by the components of health defined by the WHO (2020).

Family health is often defined by the functioning of the family (Friedman et al., 2003). Family functioning was a secondary outcome in this trial, but recent reviews of family nursing interventions have recommended it as the primary outcome measure (Østergaard et al., 2026). Family interventions may target the cognitive, affective, or behavioral domains of family functioning (Persson & Benzein, 2014; Shajani & Snell, 2023). Family involvement is conceptualized as comprising five components: family presence, participation in decision-making, receiving support, communication, and direct care (Olding et al., 2016). This framework guided how family involvement was measured in the present trial (Drakenberg et al., 2023).

A pilot study in a general intensive care setting demonstrated positive effects of face-to-face family health conversations but identified feasibility challenges related to recruitment and attrition (Ågren et al., 2019). Elective open-heart surgery shares key features with intensive care, including treatment complexity and significant emotional strain on families (Albanesi et al., 2022). However, unlike the acute and retrospective context of intensive care, elective surgery allows patients and families to prepare in advance and engage in conversations prior to the surgical event.

Building on the pilot study findings (Ågren et al., 2019), family health conversations were implemented in this study via video conferencing within the structured context of elective open-heart surgery. Face-to-face delivery was considered impractical because of travel demands before surgery and during postoperative recovery at home. Digital delivery also offered greater feasibility within clinical practice. The choice of video conferencing was further informed by a feasibility study of telephone-based conversations; the results underscored the importance of nonverbal communication (Gusdal et al., 2018), which is better supported through video interaction.

To our knowledge, this is the first study to evaluate the efficacy of family health conversations delivered via digital video conferencing. Although the intervention has been examined in other cardiovascular contexts (Gusdal et al., 2018; Østergaard et al., 2018, 2021; Rosenstrøm et al., 2022), the quantitative findings remain inconclusive, largely because of heterogeneity in intervention delivery, study design, and outcome measures (Azcárate-Cenoz et al., 2024; Østergaard et al., 2026). Moreover, qualitative research has demonstrated positive relational outcomes, including enhanced mutual understanding, improved communication, and strengthened family well-being (Azcárate-Cenoz et al., 2024; Östlund & Persson, 2014). This discrepancy between promising qualitative findings and limited quantitative evidence underscores the need for rigorous evaluation in specific clinical contexts, such as open-heart surgery.

Therefore, the aim of this study was to determine whether a family health conversation intervention, delivered via video conferencing in joint sessions with patients undergoing open-heart surgery and one or more self-identified family members, is superior to usual care in improving self-reported family well-being, functioning, and involvement among both patients and participating family members. On the basis of a pilot study (Ågren et al., 2019), we hypothesized that the mean improvement in the primary outcome operationalized through the Family Sense of Coherence scale from baseline to 90 days post hospital discharge following open-heart surgery and adjusted for baseline differences would be greater in the intervention group than in the control group. The same hypothesis was expressed regarding the secondary outcome measures of well-being, that is, health-related quality of life (Ohlsson-Nevo et al., 2021), family hardiness (Persson et al., 2016), and patient postoperative recovery (Allvin et al., 2011), and family functioning (Bylund et al., 2016). It was postulated that scores on the Family Involvement in Care Questionnaire (Drakenberg et al., 2023) would be higher in the intervention group than in the control group, as the family health conversation intervention was expected to empower family members through structured communication and increased involvement in care.

## Method

### Design

This was a two-armed, multicenter, pre–posttest superiority parallel-group randomized clinical trial comparing the effects of a family health conversation intervention in addition to usual care with those of usual care alone.

### Participants

Patients (≥18 years of age) who planned to undergo elective open-heart surgery at three university hospitals in Sweden from September 2022 to February 2024 were included in this study. Patients who were eligible for inclusion decided who belonged to their family and which family members (≥15 years of age) would be asked to participate. For inclusion in the study, one family member was required to agree to participate in the intervention. The exclusion criteria for patients and family members were that the surgery was scheduled within less than 1 month of screening, a cognitive inability to provide informed consent, or a need for a translator.

### Usual Care

Usual open-heart surgical care in Sweden does not involve any structured family support prior to surgery and does not systematically include family members in follow-up care postoperatively. Family members are allowed to visit the hospital but owing to the large catchment areas of the cardiothoracic clinics, not all family members are able to visit.

All participants, patients, and family members received usual care from health care facilities with no changes in treatment or follow-up during the study period. All patients received written practical information, addressed to the patient about the hospital stay, and were explained what to expect before, during, and after surgery. Information regarding preadmission routines, preparation, the surgical process, and recovery topics, including breathing exercises, wound care, and physical activity, was given. The purpose of the written information was to help patients feel informed, safe, and supported throughout their treatment and recovery. All patients received the telephone number of the nurses in charge of the waiting list for surgery and were informed that they could call this number if they had questions regarding their treatment. After surgery, patients were followed up in a cardiology outpatient clinic approximately 3 weeks after discharge. Usual care was directed to the patient and focused on the patient’s treatment and needs, including patient follow-up according to local and national guidelines. Although family members were allowed to accompany the patients to informational meetings, they were not systematically invited to participate. The control group answered the same questionnaires as the intervention group did.

### Intervention

In addition to receiving usual care, the intervention group received the family health conversation intervention (Benzein et al., 2008). The intervention families were offered three digital video conference conversations and a closing letter. Participation was tailored to each family’s needs, with three conversations considered standard but optional. One hour was allocated for each conversation. The patient who underwent open-heart surgery, the patient’s family, and the first author were present during the conversations. The first author is a nurse who completed a theoretical and practical third-cycle university course on family health conversations and conducted all family health conversation interventions in the study.

The conversations concerned the patients’ and family members’ beliefs about and experiences of health and illness in connection with cardiac disease and the experience of open-heart surgery. A conversation guide (Table 2) grounded in theory (Benzein et al., 2008) and based on the 12 core components of family health conversations (Östlund et al., 2015) was used for consistency in the intervention. The intervention was delivered flexibly and adapted to each family’s communicative style. For families who had difficulty expressing emotions, the nurse used reflective questions, normalization, and a paced approach, acknowledging silence or indirect expressions as meaningful communication within the Family Systems Nursing framework. In situations involving tension or differing perspectives, the nurse facilitated respectful dialog by validating multiple viewpoints and encouraging mutual reflection, aiming to promote understanding and reduce constraining beliefs rather than directly resolving conflicts. Readiness for participation was assessed by telephone after the return of the baseline assessment (prior to the first conversation) and again after the first follow-up assessment (prior to the second conversation). Participants’ expressed willingness to engage was a prerequisite for scheduling the intervention, and the third conversation was scheduled at the end of the second session.

**Table 2.** Conversation Guide for the Family Health Conversation Intervention.

Session	Main content/themes	Key questions/techniques	Notes
Applicable to all conversations	Encourage all family members to share their perceptions of their own and the family’s situation; conversation is shaped by family stories. Facilitating and hindering beliefs are identified and reflected upon. Concluding summary of the session.	Reflective questions (e.g., “When you heard your wife’s story, what were your thoughts?”); follow-up and deepening questions: “Could you tell me more about that?” “What did you think about that?” “What did you do in such a situation?”	Family members reflect on each other’s stories. Nurse guides discussion but does not provide medical advice.
First conversation	Informal introduction (“get to know each other”), discussion of purpose, structure, and expectations. Explore the family experience of the upcoming surgery and the current family situation. Creation and discussion of a genogram.	• Please describe your thoughts about the surgery? • Who in the family worries the most? • What is most important for you to talk about? • What are your preferences on (family)involvement in care?	Baseline questionnaire was not reviewed prior. Nurse introduces herself as a cardiothoracic nurse in research role. Summary of discussion at the end. The one-question question is used if appropriate. *“If you could have the answer to only one question, what would that question be?”*
Second conversation	Resource-focused session. Explore developments since the first conversation. Identify and reflect on family strengths and resources.	• What has happened since the last meeting? • What feels most relevant for you to discuss based on what you have been thinking about since the previous conversation?	Circular questions and one-question question presented if appropriate.
Third/concluding conversation	Forward-looking perspective. Discuss tools to support continued well-being and reduced suffering. Summarize all conversations and reflect on changes and significance.	Reflection on the process and outcomes of previous conversations	Family offered a closing letter 2–3 weeks after the final conversation, containing the nurse’s reflections. Intervention concluded.

The fidelity of the intervention was assured by a nurse with extensive family health conversation experience who observed and provided feedback on one session midway through the trial. No modifications were made to the intervention. The first conversation was scheduled after randomization and prior to surgery. The second conversation was scheduled after the first follow-up questionnaire was returned postoperatively, and the third conversation was held 2 weeks after the second conversation. After the conversation series, a closing letter was sent to the entire family (Bell et al., 2009; Benzein et al., 2008). As described by Benzein and colleagues, the purpose of concluding the intervention with a closing letter is for the nurse to acknowledge the family’s suffering and to offer nonjudgmental reflections on their conversations (Benzein et al., 2008).

With respect to intervention adherence, participants were considered to have received the intervention if they had attended at least one conversation session and were provided with the closing letter. Attendance was prospectively documented in a study-specific log maintained by the first author, and the number of sessions attended, as well as the dates and duration, was recorded. If a participant was unable to attend a scheduled session, the session was rescheduled when possible. When the timing of surgery prevented the first conversation from occurring prior to surgery, which occurred on nine separate occasions, the first conversation was held after the 30-day follow-up. This was not regarded as a major deviation from the protocol, as the primary outcome time point was 90 days. In cases where technical issues hindered video calls, conversations were held via telephone.

### Outcomes

The expected outcomes of family health conversations are improved family health, communal understanding, and a change in constraining beliefs about health and illness. These outcomes are achieved through cocreated alternative explanations and increased understanding and visualization of various beliefs (Persson & Benzein, 2014). Patients and their family members completed self-report paper questionnaires at baseline (before randomization) and at 30 and 90 days post discharge. Questionnaires validated in Swedish were used. The internal consistency of the scales was satisfactory at baseline, with Cronbach’s alpha values ranging from 74 to 92 for all the scales. Family well-being was measured using the Family Sense of Coherence scale, RAND-36 questionnaire, Family Hardiness Index, and Postoperative Recovery Profile questionnaire. Family functioning was measured with the General Functioning Scale, and family involvement was measured with the Family Involvement in Care Questionnaire.

### Primary Outcome

The primary outcome was the Family Sense of Coherence scale score at the 90-day follow-up. The Family Sense of Coherence measures the sense of coherence at the family level and contains 12 items answered on a 7-point Likert-type scale (Antonovsky & Sourani, 1988; Möllerberg et al., 2020). The total scale ranges from 12 to 84. Higher numbers indicate a better sense of family coherence, which is a buffer against stress (Antonovsky & Sourani, 1988). The Swedish psychometric evaluation established content validity and showed satisfactory homogeneity between items and internal consistency (α = .91) in a palliative population of patients and their family members (Möllerberg et al., 2020). The primary outcome was completed by both patients and family members.

### Secondary Outcomes

The RAND-36 questionnaire consists of eight multi-item scales that measure health-related quality of life with 35 items and one item that measures changes in health over time (Hays & Morales, 2001; Ohlsson-Nevo et al., 2021). The scales assess Physical Functioning, Role limitations due to Physical Health, Role limitations due to Emotional Problems, Energy/Fatigue, Emotional Well-being, Social Functioning, Pain, and General Health. One item represents the health transition score. The items in each scale are recalculated and then converted into scales ranging from 0 to 100, and higher numbers indicate better health-related quality of life (Hays et al., 1993). The most recent psychometric evaluation indicated satisfactory internal consistency (α > .80 for all eight multi-item scales); no floor effects were observed, although some ceiling effects were present (Ohlsson-Nevo et al., 2021).

The Family Hardiness Index measures perceived hardiness within the family and consists of 20 items answered on a 4-point Likert-type scale (Persson et al., 2016). The total scale ranges from 0 to 60, and higher numbers indicate better family well-being/togetherness. Nine questions are reverse-scored. The instrument has shown acceptable data quality, satisfactory internal consistency (α = .86), and construct validity (Persson et al., 2016).

The Postoperative Recovery Profile questionnaire is a generic measurement of postoperative recovery, consists of 19 items, and has shown good construct validity in a Swedish orthopedic and abdominal surgical setting (Allvin et al., 2011). The items are rated on a 4-point Likert-type scale. The postoperative recovery profile total scale is the sum of Items 1 to 19 and ranges from 19 to 76, with higher numbers indicating a higher level of recovery. As the original authors’ analysis instructions of the Postoperative Recovery Profile have been criticized for not fully capturing the recovery process, the total scale of the generic questionnaire was calculated and analyzed (Jakobsson, 2023). The total Postoperative Recovery Profile scale has shown satisfactory internal consistency (α = .93; Jakobsson, 2023). In addition to the generic questionnaire, nine items of a cardiac surgery-specific questionnaire were used (Bratt et al., 2017).

The General Functioning Scale is a questionnaire that measures perceived family functioning; it consists of 12 items answered on a 4-point Likert-type scale and has been psychometrically evaluated in a Swedish bariatric population (ordinal α = .92; Bylund et al., 2016). The mean score ranges from 1 to 4 and is calculated as the mean score of all 12 items. A mean score greater than two indicates unhealthy family functioning. Six items are negatively worded and require reverse scoring.

The Family Involvement in Care Questionnaire is a questionnaire that measures family involvement in inpatient care (Drakenberg et al., 2023). It consists of 16 items answered on a 4-point Likert-type scale and two open-ended questions. A “not relevant option” is also provided. The total scale ranges from 16 to 64, with higher numbers indicating greater involvement. Item 7 is reverse-scored. This instrument has been tested for content validity in a Swedish population of family members to open-heart surgical patients (Drakenberg et al., 2023).

The Family Involvement in Care Questionnaire was answered by family members only. The Postoperative Recovery Profile was completed by patients only, whereas the other secondary outcomes were completed by both patients and family members.

### Sample Size Estimation

The sample size was based on data from a pilot study (Ågren et al., 2019) in which an unpaired *t*-test was used to calculate the mean and *SD* of the Family Sense of Coherence scale score in the patient group. With a medium effect size (ES = 0.6, α = .05, 1 − β = 0.8), 116 patients needed to be randomized, with 58 in each group, to account for an anticipated dropout rate of 14%. One hundred patients were estimated to be needed for the final analysis.

### Procedures

Nurses managing surgical waitlists consecutively screened patients for eligibility and mailed two hard copies of study information and baseline questionnaires to eligible patients as they had been accepted for surgery. The distribution of study information ended when 100 families were included in the analysis. Research assistants transferred paper responses to a database via single-data entry (Garza et al., 2025) and assessed data quality at three points.

### Randomization and Blinding

Patients, along with their family members, were randomized at an allocation ratio of 1:1. Block randomization was performed, stratified according to the three hospitals. The block sizes were random, and the investigators were blinded to them. An external researcher prepared sealed envelopes according to a randomization list retrieved from an external statistician. After the participants returned the baseline questionnaires, the randomization envelope was opened by the first or last author, and the participants were informed about their allocation. No blinding was implemented: the participants and the conversation nurse (the first author) were aware of the group assignments.

### Statistical Methods

Analyses were conducted with R Statistical Software (v4.4.1, “mice,” van Buuren & Groothuis-Oudshoorn, 2011, and “glmmTMB,” Brooks et al., 2017, packages) and Statistics for Windows (v29.0.2.0). Univariate methods were used to describe participant characteristics and crude outcome data. An interim efficacy analysis was conducted after 10 families had submitted their responses to the Family Coherence scale questionnaire. No changes were made based on the interim analysis.

Multivariate Imputation by Chained Equations was used for each incomplete variable (number of imputations = 10). The data were arranged indexed by subject ID (*n* = 267) and time point (*n* = 3), resulting in 801 rows in total. Additional columns included family ID, treatment (intervention, control), subject type (patient, family member), age, gender, hospital, type of surgery, and all items from all the scales, resulting in 108 columns in total. The predictor matrix for each variable to be imputed was derived using the variable selection procedure described by van Buuren et al. (1999). Here, the prespecified minimum threshold values were 0.4 and 0.1 for the proportion of usable cases and absolute correlations, respectively. Missing data were present in scale items and the type of surgery; all other variables were complete. All scale items were treated as ordinal variables, and the proportional odds model was used as the imputation engine. For the type of surgery, polytomous regression was used as the imputation engine.

Scale scores (scaled to minimum = 0, maximum = 1) were computed for each of the scales, that is, Family Sense of Coherence, Family Hardiness Index, General Functioning Scale, Postoperative Recovery Profile, Physical Functioning, Role limitations due to Physical health, Role limitations due to Emotional problems, Energy/Fatigue, Emotional Well-being, Social Functioning, Pain, General Health, and the Health Transition score. The columns representing individual items were then discarded before the data were reshaped to a long format. Accordingly, there were two columns for the scale scores: one for baseline and the other for follow-up (indexed by subject ID, scale ID, and time).

Statistical analysis was performed using multivariate generalized linear mixed effects models with the ordered beta family (Kubinec, 2023). The parameters were estimated using maximum likelihood.

A series of competing models was fitted to the imputed data, and model selection was performed on the basis of Wald-like hypothesis tests for imputed data with α = .05 (Li et al., 1991). Full details can be found in the supporting R code, but what follows below is a description of the best-fitting model. The response variable was the follow-up score. The effect of treatment was allowed to vary by scale ID and follow-up time using a three-way interaction term. The adjustment covariates (which were all allowed to vary by scale ID using two-way interaction terms) included the baseline score, age, gender, hospital, type of surgery, and subject type. Note that including the baseline score as a predictor variable constitutes the well-established “ANCOVA” design. For interaction terms, all lower-order terms were included. All the predictors except the baseline score and age were categorical and entered into the model using dummy coding. Baseline score and age were fitted using restricted cubic splines. Subject ID and family ID were included as random effect terms (subject ID nested in family pair). Model-based marginal mean follow-up scores were computed by treatment, time, and scale ID. To quantify the treatment effect, we computed contrasts (intervention minus control) of marginal means by follow-up time and scale ID. To quantify uncertainty in our estimates of marginal means and contrasts thereof, we computed 95% confidence intervals. Accordingly, for treatment effect contrasts, confidence intervals that did not include 0 were regarded as statistically significant (α = .05).

Sensitivity analysis was conducted using three approaches to assess the potential impact of protocol deviations and the imputation of missing data (Thabane et al., 2013). Sensitivity analyses included per-protocol analysis (excluding families that missed the intervention) and two models that handled missing data differently (full imputation vs. no imputation). The results remained robust in the sensitivity analysis. Further details are available in the supporting R code.

### Ethics Approval

The study was conducted in accordance with the Declaration of Helsinki (World Medical Association, 2025) and the ethical guidelines of the Swedish Ethical Review Authority, which approved the study (Diary numbers 2019–0615; 2022-02220-02; 2022-04196-02; 2023-00788-02). All participants received comprehensive oral and written information about the purpose of the project and provided written consent. Clinical trial registration number: NCT05045196.

## Results

**Figure 1.**
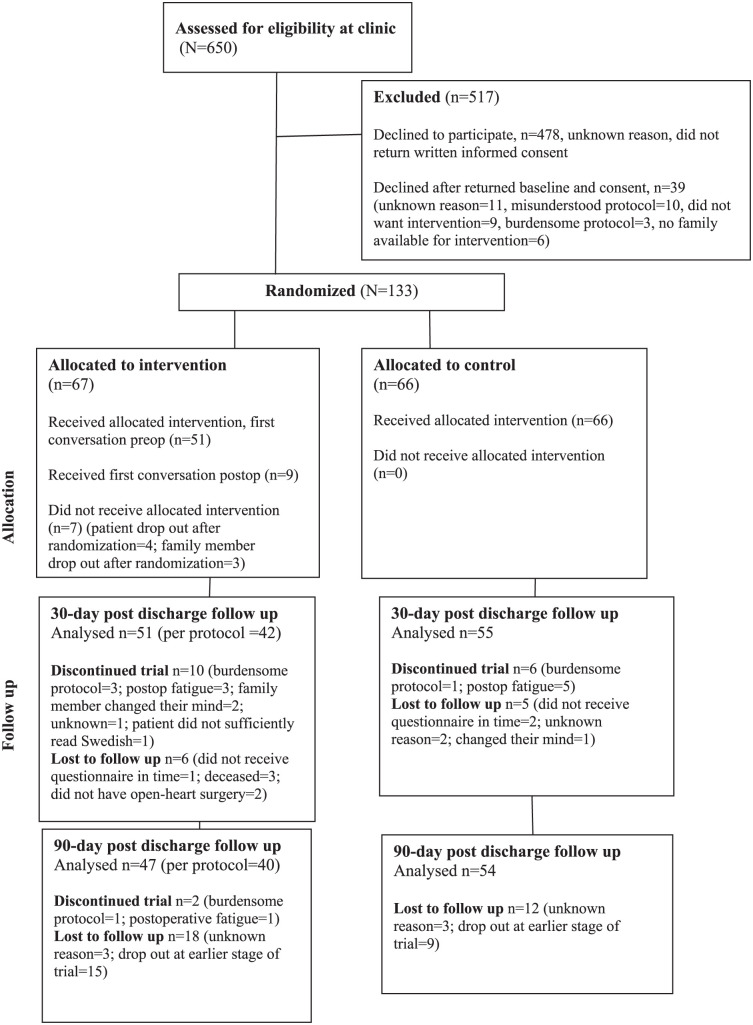
CONSORT diagram showing the flow of participating patients through each stage of the randomized trial.

**Figure 2.**
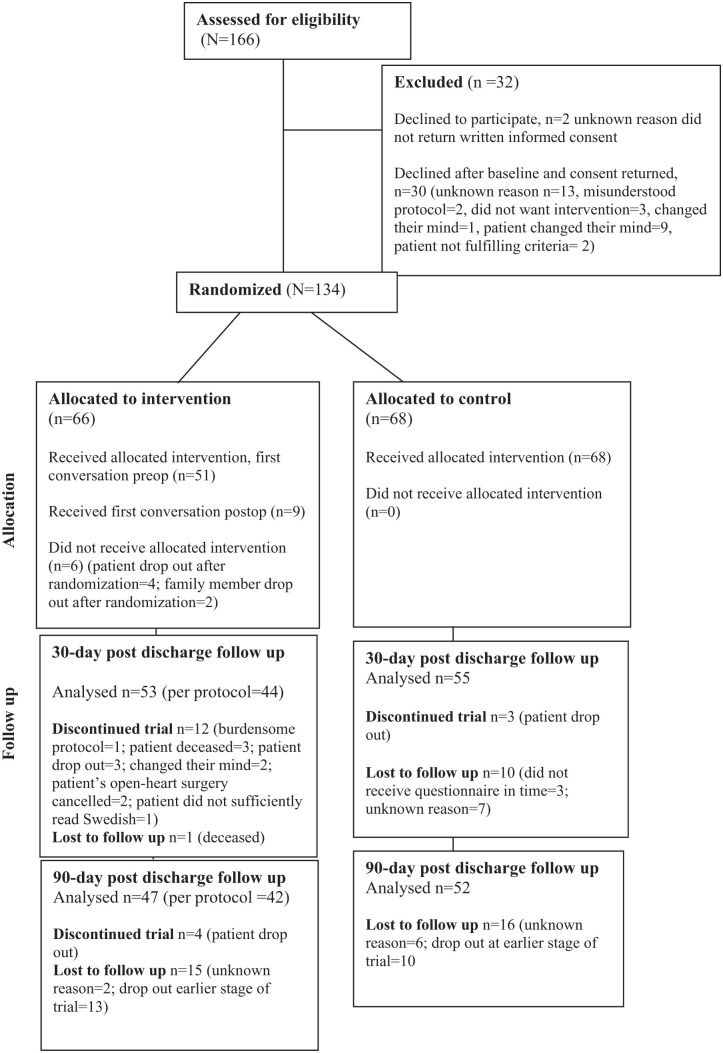
CONSORT diagram showing the flow of participating family members through each stage of the randomized trial.

Study information was mailed to 650 patients. Among these patients, 172 returned baseline data and signed consent forms. After oral information was provided by the first author, 39 patients dropped out. The reasons for dropout are presented in the CONSORT diagram (Figure 1). A total of 133 patients and 134 of their family members (Figure 2) were randomized into either the intervention group or the control group. Among the 60 families that received the intervention, 20.0% participated in one conversation, 16.7% in two conversations, and 63.3% in three conversations. The mean duration of the conversations was 51.0 min (*SD* = 14.5).

### Baseline Characteristics

The participants’ baseline characteristics were satisfactorily balanced between the groups, except for a few variables. There were some differences between the groups in terms of the level of education of the patients, and a higher percentage of adult children were included in the control group. The baseline characteristics of the participating patients and family members are presented in Table 3. The mean age of the patients was 67.1 (*SD* = 11.3) years, and 81.2% were men. Most patients had a secondary school education or higher (76.5%) and were employed (21.9%) or retired (63.8%). Single procedures were the most common type of surgery. The average age of the family members was 62.0 (*SD* = 14.3) years. The majority of family members identified as women (80.6%) and had a secondary school education or higher (91%), and most were employed (38.8%) or retired (51.5%). A comparison of the demographic information in relation to internal dropout revealed differences in the need for resternotomy among respondents (4.7%) and nonrespondents (22.2%) and differences in respondents’ (13.3%) and nonrespondents’ (18.5%) presentation of postoperative acute confusion (Supplemental Table 1). A greater percentage of adult children dropped out of the trial (22.9%) than did those who remained in the trial until the 90-day follow-up (8.1%).

**Table 3.** Self-Reported Baseline Sociodemographic Data of Randomized Patients Undergoing Open-Heart Surgery and Their Family Members.

Variables	Patients			Family members		
	Total *n* = 133	Control *n* = 66	Intervention *n* = 67	Total *n* = 134	Control *n* = 68	Intervention *n* = 66
	*M* ± *SD* (range)			*M* ± *SD* (range)		
Age	67.1 ± 11.3 (26–81)	67.5 ± 10.4 (32–81)	66.8 ± 12.2 (26–81)	62.0 ± 14.3 (23–82)	60.3 ± 14.5 (28–82)	63.8 ± 13.9 (23–82)
	*n* (%)			*n* (%)		
Gender (woman)	25 (18.8)	13 (19.7)	12 (17.9)	108 (80.6)	53 (77.9)	55 (83.3)
Education						
Primary	31 (23.3)	14 (21.2)	17 (25.4)	12 (9)	4 (5.9)	8 (12.1)
Secondary	59 (44.4)	24 (36.4)	35 (52.2)	62 (46.2)	27 (39.7)	35 (53)
University	42 (31.6)	28 (42.4)	14 (20.9)	60 (44.8)	37 (54.4)	23 (34.8)
Missing	1 (0.8)	–	1 (1.5)		–	–
Occupation						
Employed	37 (27.8)	14 (21.2)	23 (34.3)	52 (38.8)	30 (44.1)	22 (33.3)
Parental leave	1 (0.8)	1 (1.5)	–	1 (0.7)	–	1 (1.5)
Student	–	–	–	2 (1.5)	1 (1.5)	1 (1.5)
Unemployed	–	–	–	–	–	–
Retired	81 (60.9)	44 (66.7)	37 (55.2)	69 (51.5)	31 (45.6)	38 (57.6)
Sick/activity compensation	6 (4.5)	3 (4.5)	3 (4.5)	3 (2.2)	2 (2.9)	1 (1.5)
Long-term sick leave	2 (1.5)	–	2 (3)	1 (0.7)	–	1 (1.5)
Other	–	–	–	5 (3.7)	4 (5.9)	1 (1.5)
Missing	6 (4.5)	4 (6.1)	2 (3)	1 (0.7)	–	1 (1.5)
Professional experience in health care, (yes)	19 (14.3)	7 (10.6)	12 (17.9)	37 (27.6)	19 (27.9)	18 (27.3)
Feel prepared for surgery						
Yes	111 (83.5)	52 (78.8)	59 (88.1)	113 (84.3)	56 (82.4)	57 (86.4)
Missing	4 (3)	3 (4.5)	1 (1.5)	–	–	–
Cohabitating						
Yes	113 (85)	51 (77.3)	62 (92.5)	111 (82.8)	55 (80.9)	56 (84.8)
Missing	–	–	–	13 (9.7)	6 (8.8)	7 (10.6)
Family first language						
Swedish	121 (91)	59 (89.4)	62 (92.5)	124 (92.5)	62 (91.2)	62 (93.9)
Missing	4 (3)	3 (4.5)	1 (1.5)	–	–	–
Relationship to patient						
Spouse/Partner				111 (82.9)	50 (73.6)	61 (92.4)
Living apart				4 (3)	3 (4.4)	1 (1.5)
Sibling				1 (0.7)	1 (1.5)	–
Child				16 (11.9)	14 (20.6)	2 (3)
Friend				1 (.7)	–	1 (1.5)
Other				1 (0.7)	–	1 (1.5)

### Superiority of the Family Health Conversation Intervention

#### Primary Outcome

In the best-fit mixed-ordered beta model, no statistically significant effect of the intervention (point estimate proportion: 0.03, confidence interval: [−0.02, 0.07]) on the main outcome, family well-being as measured by the Family Sense of Coherence scale, was found at the 90-day follow-up.

#### Secondary Outcomes

In terms of the secondary outcomes, the intervention was superior to usual care in terms of the RAND-36 scales role limitations due to physical health, role limitations due to emotional problems, and social functioning scores at the 90-day follow-up. No statistically significant differences were detected for the other secondary outcomes that measured family well-being (physical functioning, energy/fatigue, emotional well-being, pain, general health, health transition score, and family hardness index), the Postoperative Recovery Profile, or the General Functioning scale. The proportion point estimates converted to percentage points and the reverted original scale points of the intervention are presented in Table 4 for the Family Sense of Coherence scale, Family Hardiness Index, Postoperative Recovery Profile questionnaire, General Functioning Scale, and the RAND-36 scales.

**Table 4.** Effects of the Family Health Conversation Intervention in Estimated Percentage Points and Reverted Original Scale Points at 30-Day Follow-Up and 90-Day Follow-Up.

Scale and time	Estimated points in %^a^	Cl low–up	Point estimate original scale^b^	Cl low–up	Minimum–Maximum
F-SOC					12–84
1	−1.2	−5.9 to 3.4	−0.9	−4.2 to 2.5	
2	2.7	−1.9 to 7.3	1.9	−1.4 to 5.3	
FHI					0–60
1	0.5	−4.0 to 5.1	0.3	−2.4 to 3.1	
2	3.2	−1.4 to 7.9	1.9	−0.8 to 0.2	
PRP					19–76
1	3.5	−1.5 to 8.5	2.0	−0.9 to 4.9	
2	2.0	−3.1 to 7.1	1.1	−1.8 to 4.1	
GFS					1–4
1	1.3	−3.8 to 6.4	0.0	−0.1 to 0.2	
2	−2.5	−8.3 to 3.3	−0.1	−0.3 to 0.1	
RAND-36					0-100
Physical functioning					
1	−0.5	−5.7 to 4.8			
2	1.1	−3.4 to 5.6			
Physical health^c^					
1	−7.3	−16.6 to 2.1			
2	10.9	1.8 to 20.1			
Emotional problems^d^					
1	4.7	−4.2 to 13.5			
2	17.0	8.8 to 25.1			
Energy/fatigue					
1	1.0	−4.0 to 6.0			
2	1.4	−3.5 to 6.3			
Emotional well-being					
1	0.9	−4.1 to 5.8			
2	0.5	−4.1 to 5.1			
Social functioning					
1	1.5	−4.0 to 6.9			
2	9.0	2.0 to 16.1			
Pain					
1	−2.9	−8.5 to 2.8			
2	−3.5	−9.3 to 2.2			
General health					
1	0.2	−4.4 to 4.8			
2	3.1	−1.4 to 7.6			
Health transition^e^					
1	−1.4	−6.7 to 3.9			
2	3.7	−1.8 to 9.3			

*Note.* Higher numbers on FHI scale indicate better family well-being/togetherness. Higher scores on F-SOC indicate a better sense of family coherence. GFS measures family functioning. A lower mean score indicates better family functioning, and the cutoff for unhealthy family functioning is a mean of 2. Higher scores on PRP indicate better recovery. Higher scores on RAND-36 scales indicate better health-related quality of life. Cl low–up = Confidence limit lower/upper; FHI = Family Hardiness Index; F-SOC = Family Sense Of coherence; PRP = Postoperative Recovery Profile; RAND-36 = RAND 36-item health survey 1.0; GFS = General Functioning Scale; Time 1 = 30-day follow-up; Time 2 = 90-day follow-up.

a Estimated percentage-point difference in each scale, based on the best-fitting ordinal beta model with imputed missing data. Except for the GFS, positive values indicate a benefit of the family health conversation intervention; negative values favor usual care. ^b^ Estimated difference in original scale points (converted from proportions), based on the same model. Percentage points and original scaled point estimates are the same on RAND-36 scales as they vary between 0 and 100. ^c^ Role limitations due to physical health. ^d^ Role limitations due to emotional problems. ^e^ Health transition score.

**Figure 3.**
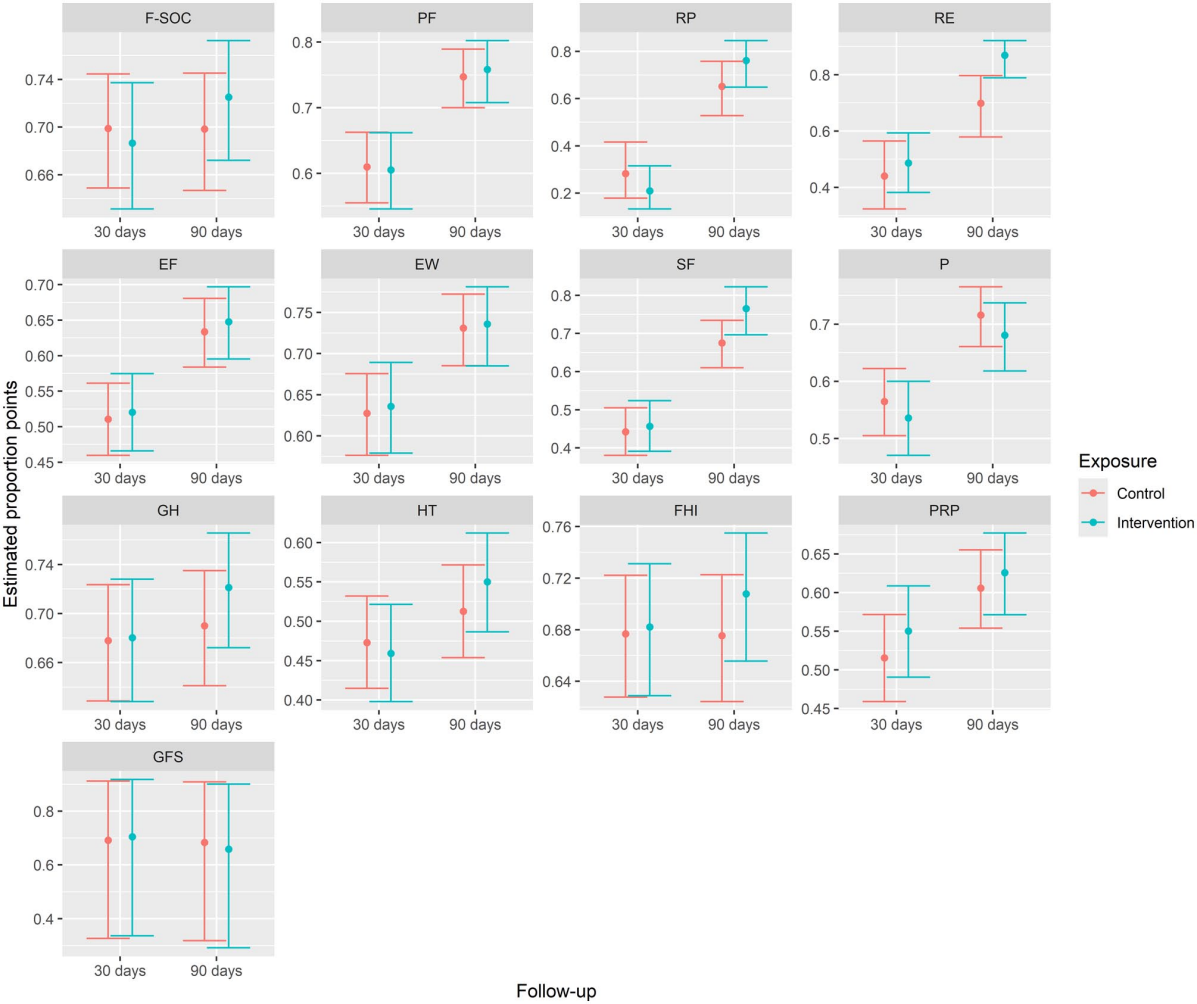
Estimated proportion point values presented with confidence intervals for each outcome variable divided into exposure groups, and presented by Time 1, 30-day follow-up, and Time 2, 90-day follow-up. *Note.* F-SOC = Family Sense of Coherence; PF = Physical Functioning; RP = Role limitations due to physical health; RE = Role limitations due to emotional problems; EF = Energy/fatigue; EW = Emotional well-being; SF = Social functioning; P = Pain; GH = General health; HT = Health Transition Score; FHI = Family Hardiness Index; PRP = Postoperative Recovery Profile; GFS = General Functioning Scale.

The estimated proportion point values are presented with confidence intervals in Figure 3 for each outcome variable included in the best-fit mixed model. The differences in estimates at 30- and 90-day follow-up between the exposure groups are shown in Figure 3. In both exposure groups, as expected, greater levels of well-being and family functioning were observed at the 90-day follow-up than at the 30-day follow-up. Compared with the control group, the intervention group had better point estimates at the 90-day follow-up except on the Pain scale of RAND-36. Family hardiness trended toward statistical significance. However, the estimated positive effect of the intervention on the Family Hardiness Index was quite low (3 percentage points at the 90-day follow-up).

#### Secondary Outcomes Not Included in the Mixed Model

Given that the Family Involvement in Care Questionnaire and the cardiac surgery-specific postoperative recovery instrument were not measured with the same interval as the other outcomes, they were excluded from the main mixed model analysis and are presented separately. The Family Involvement in Care Questionnaire revealed no clinically relevant differences in family involvement between the control (*M* = 50.4, *SD* = 6.7) and intervention (*M* = 46.7, *SD* = 7.8) groups. Notably, the complete case response rate for the Family Involvement in Care Questionnaire was small, and the descriptive data should be interpreted with caution. With respect to the mean cardiac surgery-specific postoperative recovery scores, no difference was detected at the 30-day follow-up between the exposure groups (intervention *M* = 28.8, control *M* = 28.0), and no clinically relevant difference was detected at the 90-day follow-up (intervention *M* = 30.2, control *M* = 28.5). Descriptive crude data for the baseline scores and the two follow-up scores are presented in Supplemental Table 2. No adverse effects were detected.

## Discussion

To our knowledge, this trial is the first study to evaluate the superiority of digital family health conversation interventions delivered to patients undergoing open-heart surgery and their family members. The analysis failed to detect an effect on the main outcome, family well-being as measured by the Family Sense of Coherence scale, at the 90-day follow-up. This finding may have several explanations. One possibility is that family health conversations by video conferencing are not superior to usual care in this context; another may be the relatively strong standard of usual care within the Swedish health care system, potentially creating a ceiling effect and limiting the observable additional impact of the intervention.

A third reason may be that individuals’ Family Sense of Coherence scale scores remained relatively stable over time and that more than 90 days were required for the scores to change, as identified by Åberg Petersson and colleagues (Åberg Petersson et al., 2025). Although the Family Sense of Coherence scale was selected on the basis of the theory of family health conversations (Benzein et al., 2008) and the results of a pilot study (Ågren et al., 2019), it is possible that other measurements are better suited to evaluate the effects of the family health conversations. In a secondary analysis of the effects of family health conversations in Denmark, improvements in support provided by nurses were shown (Østergaard et al., 2021). This study used the Family Functioning, Health, and Social Support instrument, which measures family functioning, health, and social support provided by nurses in one questionnaire (Østergaard et al., 2021). The Family Functioning, Health, and Social Support instrument was developed and tested for families in which one family member suffers from cardiac disease (Åstedt-Kurki et al., 2009). In addition to measuring family health and functioning, the Family Functioning, Health, and Social Support instrument (Åstedt-Kurki et al., 2009) appears to measure some of the aspects of involvement measured by the Family Involvement in Care Questionnaire (Drakenberg et al., 2023), which was used in our study. It was not possible to draw conclusions about the intervention’s impact on family involvement because of psychometric limitations. Specifically, the family members’ option to select “not relevant” had to be treated as missing data, despite being a prespecified response option. The Family Functioning, Health, and Social Support instrument could be appropriate as an alternative to the Family Involvement in Care Questionnaire in future evaluative studies of family health conversations. Family functioning being identified as a primary outcome further strengthens this (Østergaard et al., 2026). By using one instrument to capture three relevant outcomes, the risk of questionnaire burden is limited, which is a problem that was identified in previous family health conversation trials (Ågren et al., 2019; Gusdal et al., 2018). Another relevant outcome to assess would be illness beliefs, as this represents a core component of the intervention adapted to the Swedish context. Qualitative evaluation of this type of intervention is also important but was excluded from this trial in an attempt to minimize participant burden.

Some aspects of health-related quality of life were improved by the intervention in this trial. These improvements can be seen as indicators of the development of communal understanding leading to improved health, as shown in the study of Rosenstrøm and colleagues (Rosenstrøm et al., 2022). Alternatively, changes in constraining beliefs about illness could mediate improvements in health-related quality of life in relation to open-heart surgery. Like the results of Østergaard and colleagues (Østergaard et al., 2018), we detected clinically important differences in health-related quality of life subscales in favor of the family health conversation intervention. The minimal clinically important difference in the RAND-36 score is small (Hays & Morales, 2001), estimated at 3 to 5 points (Hays et al., 1993). The family health conversation intervention reduced role limitations that were affected by physical and emotional limitations and improved social functioning in a clinically important way. In contrast to our results, the previously mentioned Danish randomized clinical trial that evaluated the family health conversation intervention for heart failure in an outpatient setting revealed no effects on the total scale score for cardiomyopathy-specific health-related quality of life but did have effects on the subscale scores (Østergaard et al., 2018). This measure of health-related quality of life focuses on physical limitations due to symptom burden and social limitations and life satisfaction related to cardiomyopathy (Green et al., 2000). The differences between our results and those of the previous study may be explained by different delivery methods, contexts, and statistical analyses.

This trial was a controlled project running in parallel, as opposed to being integrated, with the health care services provided today. Future studies could benefit from patient and family involvement when pragmatic randomized clinical trials and implementation projects are planned. Such studies could be the next step for evaluating how family health conversations could be integrated into the context of open-heart surgical care. The long-term follow-up and health economic aspects of family health conversations should be studied. Consistent with the results from the pilot study (Ågren et al., 2019), the Social Functioning scale score was affected by the intervention. The RAND-36 (Ohlsson-Nevo et al., 2021) and the Family Functioning, Health, and Social Support instrument (Åstedt-Kurki et al., 2009) should be considered as primary outcomes in future evaluative family health conversation studies.

### Strengths and Limitations

The strengths of this trial were the thorough randomization procedure, the robustness of the analysis model, and the consistency of the intervention. The fidelity of the intervention was strengthened because it was delivered by the same person throughout the trial using a structured conversation guide. The lack of group allocation blinding in this trial is a common source of bias in this area of research (Østergaard et al.,2021, 2018) because it is not possible to blind participants receiving conversations in addition to usual care (Klompstra et al., 2025). Future family nursing trials should consider the use of placebo groups to reduce the risk of performance and expectation bias. A notable strength of this trial was the implementation of data quality checks, which enhanced the reliability of the data record. The external dropout rate was approximately 80% in our study, which limits the generalizability of the findings to the population of patients who receive elective open-heart surgery and might impact the ability of the trial to detect group differences. The internal dropout of patients at the first follow-up may be explained by surgery-related complications. The small sample size and the external and internal dropout rates may have limited the generalizability of the results, a problem shared with previous family health conversations trials (Ågren et al., 2019; Gusdal et al., 2018; Østergaard et al., 2018, 2021). The demographic characteristics in our study were, however, representative of the patient population demographics in terms of gender and age. The low participation rate of patients who identify as women/family members who identify as men needs special consideration in terms of study design, recruitment strategy, and patient and public involvement in future research (Kunadian et al., 2025). The consecutive recruitment in this trial favors male patients, and they constitute the majority of patients undergoing open-heart surgery. In this study, we did not have sufficient power to conduct subgroup analysis on the basis of gender, which limits the generalizability of our results to women undergoing open-heart surgery.

Furthermore, the anticipated ES was, although in line with previous research (Ågren et al., 2019; Østergaard et al., 2021), quite large considering the follow-up time and dose of the intervention, indicating a risk for a Type II error. The crude means of the RAND-36 scores in the control group in our study were in line with the crude means of the RAND-36 scores of patients who underwent percutaneous coronary intervention or coronary artery bypass grafting surgery in the first Swedish psychometric study (Orwelius et al., 2017), which indicates representativeness.

### Conclusion and Future Perspectives

The family health conversation intervention was not superior to usual care with respect to the primary outcome, family well-being as measured by the Family Sense of Coherence scale. It has the potential to improve health-related quality of life for patients and their family members. These positive effects of the family health conversations on patients’ and family members’ health-related quality of life are clinically important and suggest the need to further investigate the family health conversation intervention in real-world projects. Our findings highlight several implications for family nursing practice. Digital delivery appears feasible and may increase accessibility, but it can limit participation among patients and family members with lower technical literacy. Future interventions should focus on outcomes sensitive to changes in family functioning and involvement to more accurately capture the intervention’s impact on both patients and their families. Interventions should be further investigated with a focus on long-term effects, real-world feasibility, and appropriate outcome measures.

## Supplemental Material

sj-docx-1-jfn-10.1177_10748407261440159 – Supplemental material for Family Health Conversations—A Short-Term Supportive Intervention to Improve Family Well-Being, Functioning, and Involvement in Care After Open-Heart Surgery: A Multicenter, Randomized, Parallel-Group Superiority Trial Supplemental material, sj-docx-1-jfn-10.1177_10748407261440159 for Family Health Conversations—A Short-Term Supportive Intervention to Improve Family Well-Being, Functioning, and Involvement in Care After Open-Heart Surgery: A Multicenter, Randomized, Parallel-Group Superiority Trial by Anna Drakenberg, Daniel R. Smith, Ann-Sofie Sundqvist, Christine Leo Swenne and Elisabeth Ericsson in Journal of Family Nursing

sj-docx-2-jfn-10.1177_10748407261440159 – Supplemental material for Family Health Conversations—A Short-Term Supportive Intervention to Improve Family Well-Being, Functioning, and Involvement in Care After Open-Heart Surgery: A Multicenter, Randomized, Parallel-Group Superiority Trial Supplemental material, sj-docx-2-jfn-10.1177_10748407261440159 for Family Health Conversations—A Short-Term Supportive Intervention to Improve Family Well-Being, Functioning, and Involvement in Care After Open-Heart Surgery: A Multicenter, Randomized, Parallel-Group Superiority Trial by Anna Drakenberg, Daniel R. Smith, Ann-Sofie Sundqvist, Christine Leo Swenne and Elisabeth Ericsson in Journal of Family Nursing
