# Supplementary material for: Family Health Conversations—A Short-Term Supportive Intervention to Improve Family Well-Being, Functioning, and Involvement in Care After Open-Heart Surgery: A Multicenter, Randomized, Parallel-Group Superiority Trial
Source: J Fam Nurs. 2026 Apr 22;32(3):159–76. doi: 10.1177/10748407261440159 (PMC13291393; doi:10.1177/10748407261440159)
Supplement: sj-docx-1-jfn-10.1177_10748407261440159 – Supplemental material for Family Health Conversations—A Short-Term Supportive Intervention to Improve Family Well-Being, Functioning, and Involvement in Care After Open-Heart Surgery: A Multicenter, Randomized, Parallel-Group Superiority Trial [file sj-docx-1-jfn-10.1177_10748407261440159.docx]

| **Supplementary Table 1. Medical journal data of randomized patients, separated into included and not included groups for analysis at 30- and 90-day follow-up** | | | | |
| --- | --- | --- | --- | --- |
| Variable | Included in  30-day analysis,  n = 106 | Not included in  30-day analysis,  n = 27 | Included in  90-day analysis,  n = 101 | Not included in  90-day analysis,  n = 32 |
| Available | 105 | 17 | 101 | 21 |
| Missing | 1 | 10 | 0 | 11 |
|  | n (%) | | | |
| Preoperative disease burden |  |  |  |  |
| Arterial hypertension | 63 (59.4) | 10 (37.0) | 63 (62.4) | 10 (31.2) |
| Arrhythmia | 28 (26.4) | 6 (22.2) | 27 (26.7) | 7 (21.9) |
| Hyperlipidaemia | 23 (21.7) | 4 (14.8) | 24 (23.8) | 3 (9.4) |
| Diabetes | 14 (13.2) | 3 (11.1) | 14 (13.9) | 3 (9.38) |
| Stroke | 8 (7.6) | 2 (7.4) | 8 (7.9) | 2 (6.3) |
| Vascular disease | 2 (1.9) | – | 2 (2.0) | – |
| Chronic lung disease | 7 (6.6) | 1 (3.7) | 7 (6.9) | 1 (3.1) |
| NYHA Class |  |  |  |  |
| I | 12 (11.3) | 3 (11.1) | 12 (11.9) | 3 (9.4) |
| II | 34 (32.1) | 6 (11.1) | 32 (31.7) | 8 (25.0) |
| III | 11 (10.4) | 4 (14.8) | 11 (10.9) | 4 (12.5) |
| Surgery-related data |  |  |  |  |
| Type of surgery |  |  |  |  |
| CABG | 19 (17.9) | 3 (11.1) | 20 (19.8) | 2 (6.2) |
| Valve | 52 (49.1) | 10 (37.0) | 49 (48.5) | 13 (40.6) |
| CABG + Valve | 10 (9.4) | 1 (3.7) | 10 (9.9) | 1 (3.1) |
| Aortic | 12 (11.3) | 1 (3.7) | 12 (11.9) | 1 (3.1) |
| Aortic + Valve | 10 (9.4) | 1 (3.7) | 9 (8.9) | 2 (6.2) |
| Aortic + CABG | – | 1 (3.7) | – | 1 (3.1) |
| Other | 2 (1.9) | – | 1 (1.0) | 1 (3.1) |
|  | mean ± SD (range) | | | |
| Surgical procedure duration (hh:mm) | 3:43 ± 1:12 (1:44–8:26) | 3:45 ± 1:02 (2:10–5:50) | 3:43 ± 1:13 (1:44–8:26) | 3:41 ± 1:00 (2:02–5:50) |
| ECC duration (minutes) | 125.0 ± 52.4 (49–294) | 134.0 ± 61.5 (29–277) | 124.0 ± 53.1 (29–294) | 136.0 ± 56.1 (61–277) |
| Aortic occlusion duration | 86.8 ± 41.1 (0–236) | 92.2 ± 42.4 (17–200) | 86.1 ± 41.9 (0–236) | 94.4 ± 37.2 (37–200) |
| Priority group at surgical acceptance | n (%) | | | |
| 1, within 1-2 weeks | 1 (.9) | 1 (3.7) | 1 (.9) | 1 (3.1) |
| 2, within 1-2 months | 41 (38.7) | 7 (25.9) | 41 (40.6) | 7 (21.9) |
| 3, within 3 months) | 63 (59.4) | 9 (33.3) | 59 (58.4) | 13 (40.6) |
| Postponed surgery | 15 (15.2) | 5 (18.5) | 16 (15.8) | 4 (12.4) |
| Tertiary hospital data | mean ± SD (range) | | | |
| Length of hospital stay (days), | 7.6 ± 3.7 (4–25) | 9.4 ± 6.0 (5–27) | 7.9 ± 4.0 (4–25) | 8.0 ± 4.7 (5–27) |
|  | mean ± SD | | | |
| Readmission(s) | 7 ± 6.6 | 1 ± 3.7 | 7 ± 6.9 | 1 ± 3.1 |
| Postoperative complications | n (%) | | | |
| Postop bleed >500 ml | 57 (53.8) | 11 (40.7) | 52 (51.5) | 16 (50.0) |
| Resternotomy | 5 (4.7) | 6 (22.2) | 7 (6.9) | 4 (12.5) |
| Arrhythmia | 68 (64.2) | 9 (33.3) | 62 (61.4) | 15 (46.9) |
| Permanent pacemaker | 7 (6.6) | – | 5 (4.9) | 2 (6.3) |
| Pneumonia/VAP | – | 2 (7.4) | 1 (.9) | 1 (3.1) |
| Stroke | 1 (.9) | 2 (7.4) | 1 (1.0) | 2 (6.3) |
| Acute confusion/delirium | 14 (13.2) | 5 (18.5) | 15 (14.9) | 4 (12.5) |
| Abbreviations: CABG: coronary artery bypass grafting; ECC: extracorporeal circulation; NYHA class: New York heart association classification of heart failure; SD: standard deviation; VAP: ventilator-associated pneumonia | | | | |
