# Supplementary material for: Family Health Conversations—A Short-Term Supportive Intervention to Improve Family Well-Being, Functioning, and Involvement in Care After Open-Heart Surgery: A Multicenter, Randomized, Parallel-Group Superiority Trial
Source: J Fam Nurs. 2026 Apr 22;32(3):159–76. doi: 10.1177/10748407261440159 (PMC13291393; doi:10.1177/10748407261440159)
Supplement: sj-docx-2-jfn-10.1177_10748407261440159 – Supplemental material for Family Health Conversations—A Short-Term Supportive Intervention to Improve Family Well-Being, Functioning, and Involvement in Care After Open-Heart Surgery: A Multicenter, Randomized, Parallel-Group Superiority Trial [file sj-docx-2-jfn-10.1177_10748407261440159.docx]

| **Supplementary Table 2. Crude mean values and standard deviations (SDs) for the scales measuring patient and family member outcomes before missing data imputation at baseline, 30 days, and 90 days.** | | | | | | | | | |
| --- | --- | --- | --- | --- | --- | --- | --- | --- | --- |
| Scale | Baseline | | | 30 days | | | 90 days | | |
|  | Total | Control | Intervention | Total | Control | Intervention | Total | Control | Intervention |
| **PATIENTS** | n | Mean ± SD | | n | Mean ± SD | | n | Mean ± SD | |
| F-SOC | 127 | 72.3 ± 8.2 | 71.8 ± 10.3 | 103 | 73.8 ± 7.0 | 73.2 ± 7.5 | 98 | 73.6 ± 7.9 | 76.1 ± 5.8 |
| RAND-36^a^ |  |  |  |  |  |  |  |  |  |
| PF | 132 | 65.6 ± 23.7 | 68.8 ± 27.3 | 105 | 53.2 ± 22.8 | 59.9 ± 20.6 | 101 | 76.0 ± 19.0 | 85.3 ± 14.9 |
| RP | 132 | 37.7 ± 43.8 | 41.8 ± 43.6 | 104 | 6.0 ± 17.5 | 10.0 ± 26.2 | 98 | 47.1 ± 42.2 | 63.2 ± 41.6 |
| RE | 130 | 52.9 ± 44.2 | 62.4 ± 41.1 | 103 | 45.1 ± 43.9 | 55.1 ± 42.8 | 97 | 71.8 ± 37.0 | 84.4 ± 29.0 |
| EF | 132 | 56.7 ± 22.1 | 58.8 ± 22.9 | 105 | 47.0 ± 21.2 | 49.3 ± 20.2 | 100 | 63.8 ± 22.0 | 72.4 ± 13.8 |
| EW | 131 | 73.3 ± 18.8 | 78.0 ± 18.0 | 105 | 71.3 ± 22.8 | 79.0 ± 15.6 | 100 | 82.4 ± 14.7 | 88.1 ± 10.4 |
| SF | 133 | 68.2 ± 27.8 | 75.0 ± 24.9 | 106 | 44.5 ± 26.3 | 48.5 ± 26.1 | 101 | 77.3 ± 23.0 | 92.0 ± 15.1 |
| P | 133 | 70.6 ± 25.3 | 75.4 ± 25.6 | 106 | 53.1 ± 26.7 | 56.2 ± 25.3 | 101 | 79.4 ± 19.6 | 86.1 ± 20.7 |
| GH | 132 | 57.6 ± 20.5 | 62.3 ± 22.1 | 105 | 61.8 ± 21.3 | 71.3 ± 16.6 | 100 | 63.9 ± 20.7 | 77.6 ± 17.0 |
| HT | 132 | 33.1 ± 22.1 | 35.4 ± 23.1 | 103 | 42.6 ± 29.8 | 48.0 ± 30.5 | 101 | 57.9 ± 29.5 | 75.0 ± 26.6 |
| FHI | 127 | 47.3 ± 7.6 | 48.0 ± 6.0 | 104 | 46.1 ± 7.5 | 47.2 ± 6.6 | 97 | 47.5 ± 6.9 | 49.8 ± 6.0 |
| PRP gen^b^ | 121 | 64.6 ± 10.0 | 65.8 ± 8.1 | 98 | 59.7 ± 10.1 | 63.0 ± 6.7 | 95 | 66.9 ± 7.4 | 70.4 ± 5.2 |
| PRP thx^c^ | – | – | – | 98 | 28.0 ± 4.4 | 28.8 ± 4.4 | 90 | 28.5 ± 4.9 | 30.2 ± 4.2 |
| GFS | 129 | 1.5 ± 0.5 | 1.4 ± 0.4 | 100 | 1.5 ± 0.4 | 1.4 ± 0.4 | 96 | 1.5 ± 0.4 | 1.3 ± 0.4 |
| **FAMILY MEMBERS** | n | Mean ± SD | | n | Mean ± SD | | n | Mean ± SD | |
| FSOC | 130 | 72.3 ± 8.1 | 71.8 ± 10.2 | 104 | 70.1 ± 8.7 | 70.4 ± 10.3 | 98 | 70.4 ± 8.7 | 72.7 ± 7.7 |
| RAND-36^a^ |  |  |  |  |  |  |  |  |  |
| PF | 133 | 88.3 ± 17.6 | 84.1 ± 17.9 | 106 | 87.5 ± 19.0 | 87.3 ± 15.2 | 98 | 89.2 ± 16.7 | 86.2 ± 20.4 |
| RP | 132 | 85.3 ± 31.5 | 79.3 ± 33.8 | 105 | 79.1 ± 34.9 | 74.5 ± 32.5 | 98 | 76.4 ± 37.8 | 87.1 ± 28.4 |
| RE | 132 | 75.5 ± 38.4 | 84.4 ± 30.3 | 105 | 66.4 ± 39.1 | 66.7 ± 38.1 | 97 | 79.1 ± 34.0 | 89.1 ± 24.4 |
| EF | 129 | 65.5 ± 21.2 | 70.0 ± 19.8 | 105 | 61.7 ± 20.3 | 64.4 ± 23.3 | 99 | 69.7 ± 17.5 | 71.4 ± 16.8 |
| EW | 129 | 77.8 ± 14.7 | 79.9 ± 16.6 | 105 | 71.0 ± 16.8 | 72.6 ± 18.3 | 99 | 81.9 ± 13.8 | 83.3 ± 14.2 |
| SF | 128 | 85.2 ± 18.9 | 87.9 ± 17.7 | 105 | 72.9 ± 20.9 | 78.7 ± 21.8 | 99 | 88.9 ± 19.1 | 89.1 ± 20.0 |
| P | 128 | 81.9 ± 21.4 | 81.0 ± 20.1 | 105 | 82.9 ± 21.3 | 85.1 ± 17.1 | 99 | 82.6 ± 20.3 | 81.4 ± 19.4 |
| GH | 128 | 72.7 ± 21.7 | 71.5 ± 19.6 | 105 | 72.9 ± 21.7 | 72.1 ± 19.7 | 99 | 74.7 ± 20.7 | 73.5 ± 21.0 |
| HT | 133 | 51.1 ± 14.0 | 48.8 ± 20.9 | 106 | 51.4 ± 19.5 | 52.0 ± 20.5 | 98 | 51.0 ± 15.6 | 50.5 ± 20.1 |
| FHI | 124 | 47.7 ± 7.2 | 47.6 ± 7.4 | 103 | 47.3 ± 7.9 | 48.1 ± 7.6 | 97 | 46.3 ± 8.1 | 48.5 ± 6.7 |
| GFS | 129 | 1.4 ± 0.4 | 1.4 ± 0.4 | 104 | 1.5 ± 0.5 | 1.4 ± 0.5 | 95 | 1.5 ± 0.4 | 1.3 ± 0.4 |
| FICQ | – | – | – | 18 | 50.4 ± 6.7 | 46.7 ± 7.8 | – | – | – |
| Abbreviations and notes: FHI: Family Hardiness Index. Total scale ranges from 0–60, with higher numbers indicating better family well-being/togetherness; FICQ: family involvement in care questionnaire. The total score ranges from 16–64. A higher score indicates better family involvement. Answered by family members only; F-SOC: Family Sense of Coherence. Total scale ranges from 12-74, with higher numbers indicating a higher sense of coherence; GFS: General Functioning Scale; measures family functioning by mean calculation that varies between 1-4. A lower mean score indicates better family functioning, and the cut-off for unhealthy family functioning is a mean of 2; PRP: Postoperative RecoveryPprofile; RAND-36: RAND 36-item health survey 1.0.  ^a^RAND-36 scales: Physical functioning (PF), role limitations due to physical health (RP), role limitations due to emotional problems (RE), energy/fatigue (EF), emotional well-being (EW), social functioning (SF), pain (P), general health (GH), and the health transition (HT) score. When the score ranges from 0–100, higher scores indicate better health-related quality of life.^b^PRP generic total scale ranges from 19–76; higher numbers indicate lower symptom burden/better recovery.^c^PRP cardiac surgery-specific items; total scale ranges from 9-36, where higher numbers indicate lower symptom burden/better recovery. No items are answered at baseline as they are only relevant postoperatively. PRP is provided by patients only. | | | | | | | | | |
